# Hidden Fracture, Urgent Thrombolysis: Intravenous Alteplase for Acute Ischemic Stroke With an Occult Proximal Femoral Fracture After a Fall

**DOI:** 10.7759/cureus.110783

**Published:** 2026-06-13

**Authors:** Tatsuya Tanaka, Takashi Furukawa, Taku Goto, Hiroko Nakashiro, Yukinori Takase, Akira Matsuno

**Affiliations:** 1 Department of Neurosurgery, International University of Health and Welfare Narita Hospital, Narita, JPN; 2 Department of Neurosurgery, Takagi Hospital, Okawa, JPN; 3 Department of Emergency Medicine, Takagi Hospital, Okawa, JPN

**Keywords:** acute ischemic stroke, extracranial bleeding, intravenous tissue plasminogen activator, occult proximal femoral fracture, onset-associated fall, thrombolysis, trauma assessment

## Abstract

An 89-year-old woman with chronic atrial fibrillation fell to the right side and was found to have right-sided weakness and impaired consciousness. Initial evaluation, including Focused Assessment with Sonography for Trauma (FAST), head computed tomography (CT), cervical CT, and thoracoabdominal CT, showed no intracranial hemorrhage, cervical epidural hematoma, aortic dissection, or thoracoabdominal bleeding. Brain magnetic resonance imaging (MRI) showed acute ischemic lesions in the left middle cerebral artery territory with a diffusion-weighted imaging-fluid-attenuated inversion recovery (DWI-FLAIR) mismatch. Intravenous tissue plasminogen activator (IV tPA) was administered for acute ischemic stroke. After IV tPA initiation, retrospective review of the initial thoracoabdominal CT revealed an occult right proximal femoral fracture on the paretic side. Although no intracranial hemorrhage occurred, the patient developed right lower limb swelling and progressive anemia requiring red blood cell transfusion. This case illustrates that proximal femoral fractures may be overlooked in patients with acute ischemic stroke presenting after a fall, particularly when pain localization and voluntary limb movement are impaired. Systematic evaluation of the pelvis, hip, and proximal femur before thrombolysis, followed by careful monitoring for extracranial bleeding after treatment, is important.

## Introduction

Intravenous tissue plasminogen activator (IV tPA) is a standard reperfusion therapy for acute ischemic stroke and is intended to improve neurological outcomes when administered early after symptom onset [1,2]. However, because this treatment carries a risk of hemorrhagic complications, not only intracranial hemorrhage but also potential extracranial bleeding sources should be carefully assessed before treatment [1,2]. In particular, patients with acute ischemic stroke accompanied by an onset-associated fall may have concomitant traumatic injuries [3]. Because impaired consciousness, aphasia, and hemiparesis can obscure the presence and localization of pain, fractures or truncal injuries may be overlooked.

In determining eligibility for IV tPA, “major surgery or serious non-head trauma within 14 days” is an important exclusion criterion [1,2]. However, in clinical practice, the precise scope of “serious trauma” is not always clear, and clinicians must rapidly weigh the risk of bleeding against the expected benefit of reperfusion therapy within a limited time frame.

We report the case of an 89-year-old woman with acute cardiogenic cerebral embolism who presented after a fall to the right side and was found to have right-sided hemiparesis and impaired consciousness. An occult right proximal femoral fracture was identified after IV tPA initiation, followed by progressive anemia. We discuss the challenges of evaluating occult serious extracranial trauma during time-critical thrombolysis assessment in patients with acute ischemic stroke presenting after a fall.

## Case presentation

The patient was an 89-year-old woman with a history of hypertension, chronic heart failure, chronic atrial fibrillation, and anemia. Before onset, she was able to walk with a cane, and her modified Rankin Scale (mRS) score [4] was three. She was not taking any anticoagulant or antiplatelet agents.

On day zero, she had been attending a day-care facility since 9:00 a.m. At approximately 9:50 a.m., she fell to her right side. When the facility staff evaluated her, right upper and lower limb weakness was noted, and acute stroke was suspected. She was transported to our hospital and arrived at 10:35 a.m.

On arrival, her body temperature was 36.6°C (reference range: 36.5-37.5°C), heart rate was 67 beats/min (reference range: 60-100 beats/min) with an irregular rhythm, blood pressure was 100/60 mmHg, oxygen saturation was 97% on room air (reference: ≥95%), and respiratory rate was 20 breaths/min (reference range: 12-20 breaths/min). Her level of consciousness was Glasgow Coma Scale (GCS) E4V2M5. Focused Assessment with Sonography for Trauma (FAST) showed no findings suggestive of thoracoabdominal hemorrhage, and no obvious active external bleeding was observed. There was no anisocoria, and facial palsy and dysarthria were not apparent. Neurologically, manual muscle testing showed decreased strength of the right upper and lower limbs, graded 2/5, whereas movement of the left upper and lower limbs was preserved.

Laboratory findings on arrival are summarized in Table 1.

**Table 1:** Laboratory findings on arrival

Parameter	Result	Reference range	Unit
White blood cell count	5,540	3,500–9,700	/μL
Red blood cell count	314	376–516	×10⁴/μL
Hemoglobin	8.6	11.2–15.2	g/dL
Hematocrit	27.2	34.3–45.2	%
Platelet count	26.8	14.0–37.9	×10⁴/μL
Prothrombin time-international normalized ratio	1.01	0.80–1.20	—
Activated partial thromboplastin time	24.3	24.0–34.0	seconds
Fibrinogen	313	160–360	mg/dL
D-dimer	4.4	≤1.0	μg/mL
Glucose	102	69–109	mg/dL
Hemoglobin A1c	5.6	4.6–6.2	%
Blood urea nitrogen	15	8–22	mg/dL
Creatinine	1.01	0.40–0.70	mg/dL
Estimated glomerular filtration rate	39		mL/min/1.73 m²
Sodium	139	138–146	mEq/L
Potassium	3.6	3.6–4.9	mEq/L
Chloride	102	99–109	mEq/L
Total protein	6.9	6.7–8.3	g/dL
Albumin	3.6	4.0–5.0	g/dL
Total bilirubin	0.3	0.3–1.2	mg/dL
Aspartate aminotransferase	16	13–33	U/L
Alanine aminotransferase	5	6–30	U/L
Alkaline phosphatase	63	38–113	U/L
Lactate dehydrogenase	196	124–222	U/L
γ-glutamyl transpeptidase	18	10–47	U/L
C-reactive protein	0.07	≤0.20	mg/dL
Brain natriuretic peptide	354	0.0–18.4	pg/mL

The patient had anemia at presentation, with a hemoglobin level of 8.6 g/dL and a hematocrit of 27.2%. The platelet count was 26.8 × 10⁴/μL, the prothrombin time-international normalized ratio was 1.01, and the activated partial thromboplastin time was 24.3 seconds, indicating no thrombocytopenia or marked coagulation prolongation that would preclude intravenous thrombolysis. The glucose level was 102 mg/dL and the hemoglobin A1c level was 5.6%, indicating no marked glucose abnormality at presentation. Liver enzyme levels were not elevated, whereas the albumin level was mildly decreased at 3.6 g/dL. The D-dimer level was elevated at 4.4 μg/mL, and the brain natriuretic peptide level was elevated at 354.0 pg/mL.

After arrival, an initial evaluation was performed, including assessment for fall-related trauma and thoracoabdominal major vascular disease. Head CT, cervical CT, and thoracoabdominal CT were initiated at 10:38 a.m. Head CT showed no intracranial hemorrhage or obvious early ischemic change (Figure 1).

**Figure 1:**
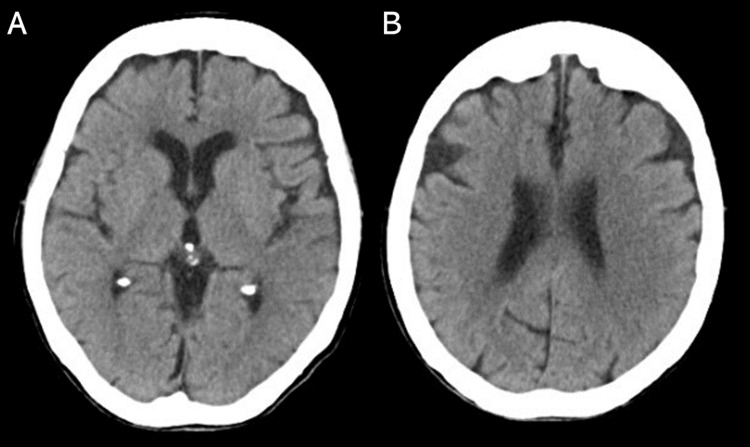
Non-contrast head computed tomography on arrival Axial non-contrast head computed tomography images showed no intracranial hemorrhage, obvious early ischemic change, or traumatic intracranial lesion.

Cervical CT showed no obvious cervical spine fracture or findings suggestive of cervical epidural hematoma within the spinal canal (Figure 2A).

**Figure 2:**
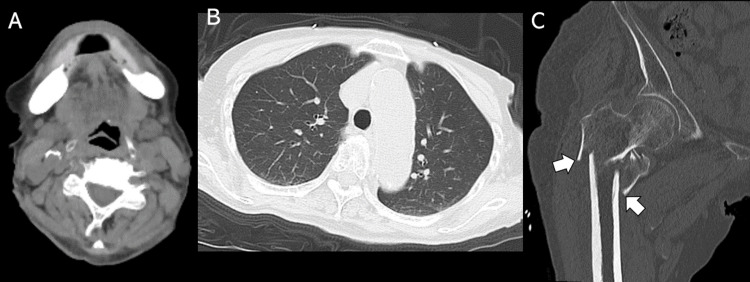
Computed tomography assessment before intravenous tissue plasminogen activator administration (A) Axial cervical computed tomography showed no cervical spine fracture or cervical epidural hematoma; (B) Axial chest computed tomography showed no evidence of aortic arch dissection, hemothorax, pneumothorax, mediastinal hematoma, or obvious traumatic thoracic injury; (C) Coronal bone-window computed tomography image of the right hip, retrospectively reviewed after intravenous tissue plasminogen activator initiation, showed a comminuted fracture extending from the right trochanteric region to the subtrochanteric femur (white arrows).

Thoracoabdominal CT showed no findings suggestive of aortic dissection, including the aortic arch, and no hemothorax, pneumothorax, mediastinal hematoma, intra-abdominal hematoma, or obvious visceral injury (Figure 2B). No obvious acute traumatic fracture of the trunk was suspected. The right proximal femur was included within the scan range of the thoracoabdominal CT obtained on arrival, and retrospective review showed a comminuted fracture extending from the right trochanteric region to the subtrochanteric femur; however, this fracture was not recognized before initiation of IV tPA therapy (Figure 2C). Electrocardiography showed atrial fibrillation (Figure 3).

**Figure 3:**
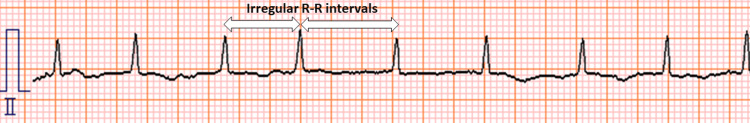
Electrocardiography on arrival Lead II rhythm strip showed atrial fibrillation, characterized by irregularly irregular R-R intervals and the absence of consistent preceding P waves. Double-headed arrows indicate variable R-R intervals.

Brain magnetic resonance imaging (MRI) was started at 11:03 a.m. Diffusion-weighted imaging (DWI) showed a hyperintense lesion in the posterior M2 branch territory of the left middle cerebral artery (MCA), whereas fluid-attenuated inversion recovery (FLAIR) imaging showed no obvious signal change, suggesting hyperacute cerebral infarction with a DWI-FLAIR mismatch (Figures 4A-4D).

**Figure 4:**
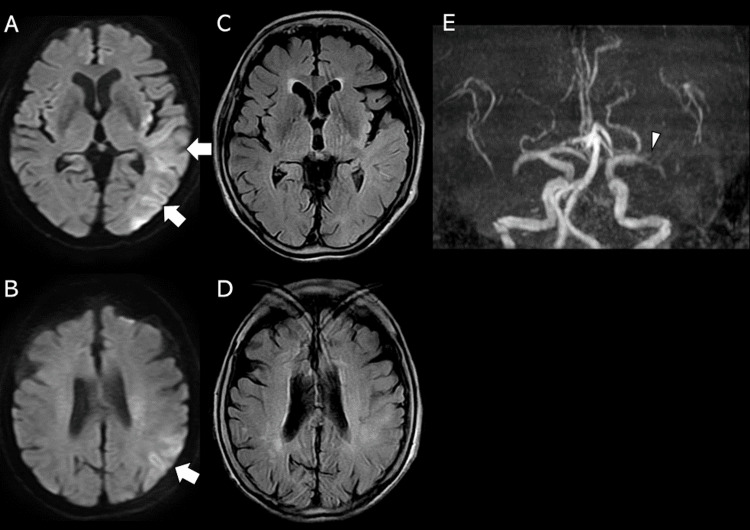
Magnetic resonance imaging and angiography before IV tPA administration (A and B) Diffusion-weighted imaging (DWI) showed acute ischemic lesions in the left middle cerebral artery territory, mainly in the left temporoparietal region (white arrows); (C and D) Fluid-attenuated inversion recovery (FLAIR) imaging showed no corresponding hyperintense signal change, indicating a DWI-FLAIR mismatch; (E) Magnetic resonance angiography (MRA) showed poor visualization of the distal left middle cerebral artery branches, suggesting M2 stenosis or occlusion (white arrowhead). IV tPA: Intravenous tissue plasminogen activator.

Magnetic resonance angiography (MRA) suggested stenosis or occlusion distal to the left MCA M2 segment (Figure 4E). After MRI, the patient complained of pain; however, the location of the pain was unclear.

Based on these findings, cardiogenic cerebral embolism associated with chronic atrial fibrillation was diagnosed. DWI-FLAIR mismatch suggested hyperacute ischemic stroke and supported the indication for intravenous thrombolysis. However, MRA did not show a definite proximal large-vessel occlusion, such as internal carotid artery terminus, M1, or proximal M2 occlusion. Considering the distal location of the suspected occlusion, the patient’s advanced age and prestroke mRS score, and the potential risk of procedure-related hemorrhagic complications, mechanical thrombectomy was not pursued. Based on the time of onset, imaging findings, and clinical symptoms, IV tPA was initiated at 11:48 a.m. at the standard Japanese dose of 0.6 mg/kg. The onset-to-needle time was 118 minutes, and the door-to-needle time was 73 minutes.

After initiation of IV tPA, review of the thoracoabdominal CT obtained on arrival revealed a comminuted fracture extending from the right trochanteric region to the subtrochanteric femur within the scan range. At the time the fracture was identified, there was no apparent decrease in blood pressure or deterioration in respiratory status, and the scheduled dose of IV tPA was completed. No obvious deterioration in vital signs was observed after admission. The clinical timeline from onset to transfer is summarized in Table 2.

**Table 2:** Clinical timeline The timeline summarizes the acute clinical course from the onset-associated fall to the initial emergency assessment, imaging studies, intravenous thrombolysis, and subsequent recognition of the occult proximal femoral fracture. CT: computed tomography; DWI: diffusion-weighted imaging; FLAIR: fluid-attenuated inversion recovery; GCS: Glasgow Coma Scale; IV: intravenous; MCA: middle cerebral artery; MRA: magnetic resonance angiography; MRI: magnetic resonance imaging; tPA: tissue plasminogen activator; GCS E4V2M5 indicates eye opening 4, verbal response 2, and motor response 5.

Time / Day	Event	Findings / Management
9:00 a.m.	Attendance at day-care facility	
9:50 a.m.	Fall to the right side	Right-sided weakness was noted
10:35 a.m.	Arrival at hospital	GCS E4V2M5; right upper and lower limb weakness
10:38 a.m.	CT evaluation	No intracranial hemorrhage; no cervical epidural hematoma; no thoracoabdominal bleeding
11:03 a.m.	Brain MRI/MRA	Acute ischemic lesion in the left MCA territory with DWI-FLAIR mismatch
11:48 a.m.	IV tPA initiation	The onset-to-needle time was 118 minutes, and the door-to-needle time was 73 minutes.
After IV tPA initiation	Retrospective CT review	Occult right proximal femoral fracture identified

Post-IV tPA brain imaging showed no intracranial hemorrhage. However, swelling, warmth, and pain of the right lower limb were observed. On day one, the hemoglobin level decreased to 6.8 g/dL, and two units of packed red blood cells were transfused. On day one, the hemoglobin level was 7.8 g/dL, and an additional two units of packed red blood cells were transfused. The progression of anemia was considered multifactorial, potentially involving local bleeding associated with the femoral fracture, the effect of IV tPA, pre-existing anemia, low body weight, and chronic heart failure.

On day one, after confirming the absence of intracranial hemorrhage on post-IV tPA brain imaging, apixaban 2.5 mg twice daily was started for secondary prevention. The right proximal femoral fracture was managed by the orthopedic surgery team, and osteosynthesis was performed on day 22 (Figure 5).

**Figure 5:**
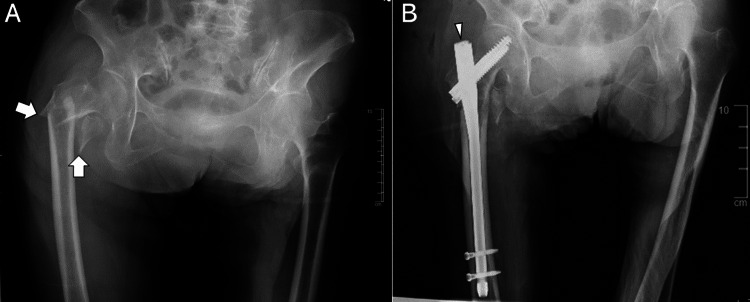
Right proximal femoral fracture before and after osteosynthesis (A) Anteroposterior pelvic radiograph showed a comminuted fracture of the right proximal femur extending from the trochanteric region to the subtrochanteric area (white arrows); (B) Postoperative radiograph showed internal fixation with a cephalomedullary nail (white arrowhead).

The postoperative course was uneventful.

Rehabilitation was continued; however, at transfer, her level of consciousness was GCS E4V4M6. Although no obvious quadriparesis was observed, generalized muscle weakness was marked, and mild right upper and lower limb weakness remained. She also had prominent sensory aphasia and required nutritional management via a nasogastric tube. Her activities of daily living remained bed-level, and she was transferred to the referring hospital on day 78 for continued inpatient care. Her mRS score at transfer was five.

## Discussion

This case involved an 89-year-old woman with acute cardiogenic cerebral embolism after an onset-associated fall. After IV tPA therapy was administered, a right proximal femoral fracture that had been included within the scan range of the CT performed on arrival was identified. This case illustrates an important clinical challenge in acute ischemic stroke after an onset-associated fall: balancing the time-sensitive need for reperfusion therapy with the evaluation of occult serious extracranial trauma.

In determining eligibility for IV tPA, “major surgery or serious non-head trauma within 14 days” is considered an important exclusion criterion [1,2]. However, the precise scope of “serious trauma” is not always clearly defined in clinical practice. A proximal femoral fracture may cause progressive anemia due to bleeding into the surrounding soft tissue and can become a clinically important extracranial bleeding source [5,6], particularly under thrombolytic therapy. In the present case, the fracture was included within the scan range of the initial CT but was not recognized before IV tPA initiation. In retrospect, this fracture could be regarded as a condition potentially meeting the criterion of “serious non-head trauma.” If the proximal femoral fracture had been recognized before IV tPA administration, the indication for thrombolysis would have required careful reassessment because the fracture could represent a significant extracranial bleeding source. Recognition of this injury might have led us to withhold IV tPA or reconsider its use after weighing the expected neurological benefit against the risk of fracture-site bleeding. This case should not be interpreted as supporting routine thrombolysis in patients with proximal femoral fractures, but rather as highlighting the importance of identifying such injuries before treatment and incorporating them into the risk-benefit assessment.

In this case, FAST, cervical CT, and thoracoabdominal CT were performed on arrival to evaluate potentially critical contraindications to IV tPA, including intracranial hemorrhage, cervical epidural hematoma, aortic dissection, and thoracoabdominal bleeding. However, the right proximal femoral fracture was not recognized during the time-critical initial evaluation. This represents an important lesson: in patients with acute stroke after an onset-associated fall, clinicians should evaluate not only for life-threatening head, neck, thoracic, and abdominal conditions, but also for injuries involving the pelvis, hip, and proximal femur.

Trauma evaluation can be particularly difficult in patients with acute ischemic stroke because hemiparesis, impaired consciousness, aphasia, or higher cortical dysfunction may prevent patients from reporting pain adequately or localizing it clearly [3]. Shirokane et al. reported hemothorax caused by initially unrecognized rib fractures after tPA administration for acute ischemic stroke and emphasized the importance of evaluating thoracic trauma before thrombolysis [3]. In addition, fractures after stroke have been reported to occur more frequently on the paretic side, probably because of falls toward the paretic side, sensory impairment, impaired balance, reduced protective responses, and decreased bone mineral density [7]. Therefore, when a patient falls at the onset of acute ischemic stroke, limb weakness on the paretic side should not be attributed solely to stroke symptoms; concomitant traumatic injuries, including ipsilateral hip and proximal femoral fractures, should also be considered.

IV tPA therapy should not be uniformly withheld in all patients with possible trauma. Dressing et al. reported successful tPA therapy without major hemorrhagic complications in a patient with acute ischemic stroke and rib fractures after traumatic cardiopulmonary resuscitation, following careful risk-benefit assessment [8]. Thus, although trauma may increase bleeding risk, the potential benefit of reperfusion therapy should be individually weighed against that risk. The present case should not be interpreted as showing that IV tPA can be safely administered despite a fracture. Rather, it demonstrates that occult serious trauma should be evaluated as systematically as possible before treatment and that extracranial bleeding should be carefully monitored after IV tPA administration.

A proximal femoral fracture in the acute phase of ischemic stroke can affect not only bleeding risk but also functional outcome. Yuan et al. reported that patients with acute cerebral infarction complicated by acute hip fracture had worse neurological outcomes, longer hospital stays, and higher rates of pneumonia, deep vein thrombosis, and one-year mortality than patients with acute cerebral infarction alone [9]. In the present case, although no obvious progression of cerebral infarction or intracranial hemorrhage was observed, the mRS score at transfer was five. The proximal femoral fracture may have affected the clinical course through restriction of early mobilization, delayed rehabilitation, and systemic complications.

Premorbid clinical frailty may also have contributed to the clinical course and poor functional outcome in this case. Although the Clinical Frailty Scale was not formally assessed, the patient had several frailty-related features, including advanced age, cane-assisted ambulation, day-care attendance, a prestroke mRS score of three, chronic heart failure, anemia, and mildly decreased albumin levels. Pedregosa et al. reported that premorbid frailty was associated with poorer functional outcomes and higher 30-day mortality after intravenous thrombolysis in older patients with acute ischemic stroke, although not with symptomatic intracerebral hemorrhage [10]. In this case, reduced physiologic reserve related to frailty may have limited recovery after acute ischemic stroke and subsequent complications. Frailty should therefore be considered when estimating prognosis, although it should not be regarded as an absolute contraindication to intravenous thrombolysis.

A practical approach in such patients may include two rapid steps before thrombolysis. First, clinicians should confirm the mechanism and side of the fall and perform a focused trauma examination, including inspection and palpation of the pelvis, hip, and proximal femur for pain, swelling, deformity, shortening, or external rotation. Second, when CT is performed to exclude head, cervical, thoracic, or abdominal contraindications, the CT scout images, lower edge of the scan range, and bone-window images should be reviewed specifically for pelvic, hip, and proximal femoral fractures. If pain localization is unreliable because of aphasia, impaired consciousness, or hemiparesis, or if the patient has fallen toward the paretic side, additional pelvic or hip radiography or dedicated CT should be considered, provided that it can be performed without substantially delaying reperfusion therapy. After IV tPA administration, clinicians should continue monitoring for extracranial bleeding, including fracture-site bleeding and progression of anemia, in addition to intracranial hemorrhage.

## Conclusions

In patients with acute ischemic stroke accompanied by an onset-associated fall, the localization of pain may be unclear because of hemiparesis, aphasia, or impaired consciousness. As a result, serious non-head trauma, such as a proximal femoral fracture, may be overlooked during the initial emergency evaluation. When considering IV tPA therapy, systematic trauma assessment is important in addition to excluding intracranial hemorrhage. Particular attention should be paid to the pelvis, hip, and proximal femur, especially in older patients or those who cannot clearly report pain. After thrombolysis, careful monitoring is required not only for intracranial hemorrhage but also for fracture-site bleeding, soft-tissue hemorrhage, and progression of anemia. This case emphasizes the need to balance the urgency of reperfusion therapy with careful assessment for occult traumatic injury after a fall.
